# Text4Heart II – improving medication adherence in people with heart disease: a study protocol for a randomized controlled trial

**DOI:** 10.1186/s13063-018-2468-z

**Published:** 2018-01-25

**Authors:** Ralph Maddison, Ralph Stewart, Rob Doughty, Tony Scott, Andrew Kerr, Jocelyne Benatar, Robyn Whittaker, Jonathan C. Rawstorn, Anna Rolleston, Yannan Jiang, Paul Estabrooks, Rachel Karen Sullivan, Hannah Bartley, Leila Pfaeffli Dale

**Affiliations:** 1 Institute for Physical Activity and Nutrition, Deakin University, Geelong, VIC Australia; 2 Department of Cardiology, Auckland District Health Board, Auckland, New Zealand; 3 Heart Health Research Group, Department of Medicine, University of Auckland, Auckland, New Zealand; 4 Department of Cardiology, Waitemata District Health Board, Auckland, New Zealand; 5 Epidemiology and Biostatistics, University of Auckland, Auckland, New Zealand; 6 National Institute for Health Innovation, School of Population Health, University of Auckland, Auckland, New Zealand; 7 The Centre for Health, Tauranga, New Zealand; 8 Department of Health Promotion, Social and Behavioral Health, University of Nebraska Medical Centre, Omaha, NE USA; 9 Department of Exercise Sciences, Faculty of Science, University of Auckland, Auckland, New Zealand; 10 School of Kinesiology, University of British Columbia, Vancouver, BC Canada

**Keywords:** Cardiovascular disease, Self-management, Text messaging, Risk factors

## Abstract

**Background:**

Cardiac rehabilitation (CR) is an essential component of contemporary management for patients with coronary heart disease, including following an acute coronary syndrome (ACS). CR typically involves education and support to assist people following an ACS to make lifestyle changes and prevent subsequent events. Despite its benefits, uptake and participation in tradition CR programs is low. The use of mobile technologies (mHealth) offers the potential to improve reach, access, and delivery of CR support. We aim to determine the effectiveness and cost-effectiveness of a text-messaging intervention (Text4Heart II) to improve adherence to medication and lifestyle change in addition to usual care in people following an ACS. A second aim is to use the RE-AIM framework to inform the potential implementation of Text4Heart II within health services in New Zealand.

**Methods:**

Text4Heart II is a two-arm, parallel, superiority randomized controlled trial conducted in two large metropolitan hospitals in Auckland, New Zealand. Three hundred and thirty participants will be randomized to either a 24-week theory- and evidence-based personalized text message program to support self-management in addition to usual CR, or usual CR alone (control). Outcomes are assessed at 6 and 12 months. The primary outcome is the proportion of participants adhering to medication at 6 months as measured by dispensed records. Secondary outcomes include medication adherence at 12 months, the proportion of participants adhering to self-reported healthy behaviors (physical activity, fruit and vegetable consumption, moderating alcohol intake and smoking status) measured using a composite health behavior score, self-reported medication adherence, cardiovascular risk factors (lipids, blood pressure), readmissions and related hospital events at 6 and 12 months. A cost-effectiveness analysis will also be conducted. Using the RE-AIM framework, we will determine uptake and sustainability of the intervention.

**Discussion:**

The Text4Heart II trial will determine the effectiveness of a text-messaging intervention to improve adherence to medication and lifestyle behaviors at both 6 and 12 months. Using the RE-AIM framework this trial will provide much needed data and insight into the potential implementation of Text4Heart II. This trial addresses many limitations/criticisms of previous mHealth trials; it builds on our Text4Heart pilot trial, it is adequately powered, has sufficient duration to elicit behavior change, and the follow-up assessments (6 and 12 months) are long enough to determine the sustained effect of the intervention.

**Trial registration:**

Australian New Zealand Clinical Trials Registry, ID: ACTRN12616000422426. Registered retrospectively on 1 April 2016.

**Electronic supplementary material:**

The online version of this article (10.1186/s13063-018-2468-z) contains supplementary material, which is available to authorized users.

## Background

Cardiovascular diseases (CVD) are the leading causes of premature death and disability worldwide, accounting for 30% of all global deaths [1]. By 2030, almost 23.6 million people will die from CVD, mainly coronary heart disease (CHD), and the total number of disability-adjusted life years attributable to CVD is expected to reach approximately 204 million [1]. People with CVD are more likely to develop future cardiac events such as unstable angina, myocardial infarction (MI), and sudden cardiac death [2]. This places a huge burden on healthcare systems, with the US spending US$444 billion on CVD-related treatments in 2010 [3].

Improved diagnosis, treatment, and management have substantially reduced the mortality rate of individuals living with CHD [4, 5]; however, those who have experienced an MI have a 20–40% risk of a recurrent event or death in the next 5 years [4, 5]. Approximately 80% of CHD is caused by modifiable risk factors including physical inactivity, smoking, unhealthy diet, and harmful alcohol consumption [6], and effective evidence-based secondary prevention treatments—such as implementing lifestyle changes and adhering to prescribed medication regimens (self-management)—can aid recovery and reduce recurrent cardiac events. Improvements in lipids, systolic blood pressure, smoking prevalence, and physical activity account for an estimated 47% of the total improvement in case fatality [5].

### Management of CHD

Acute coronary syndrome (ACS) encompasses unstable angina, and MI. Following diagnosis of an ACS, patients should receive a range of evidence-based preventive treatments that include appropriate clinical follow-up, as well as referral to programs that provide education and support self-management for the secondary prevention of disease—commonly known as cardiac rehabilitation (CR). A core focus of CR is to encourage people to make healthy lifestyle changes to reduce subsequent cardiac events. Lifestyle behavior changes include regular physical activity, eating a healthy diet, stopping smoking, reducing harmful alcohol intake, and taking medications as per a prescribed regimen [7]. Empowering self-management is critical for people with an ACS to maximize treatment benefits [8].

CR is an essential part of contemporary management for people with an ACS. It has been shown to reduce cardiovascular deaths and hospital readmissions by 25% [7], and is cost-effective for those who participate [9]. A Cochrane systematic review [10] of CR reported a statistically significant reduction in cardiovascular mortality of 26% (odds ratio (OR) 0.74, 95% CI 0.57 to 0.96) and a reduction in all-cause mortality of 13% (OR 0.87, 95% confidence interval (CI) 0.71 to 1.05). This magnitude of effect is consistent with a review of cohort studies and randomized controlled trials (RCTs) [11].

Despite these benefits, CR participation is inadequate in all countries in which it has been assessed [12]. Low levels of patient participation and completion (14–43% after MI) have been reported in Australia, France, the United Kingdom, and New Zealand with high levels of dropout after enrollment [13–17]. Lack of completion reduces the benefits of CR, such as improvements in CVD risk factors [18]. Patient-oriented, medical, and healthcare system factors associated with suboptimal participation include availability, affordability and accessibility of a program, as well as work/domestic commitments and psychological barriers [19, 20]. Current CR delivery approaches do not suit all people and new models are needed to improve the uptake and completion of CR. A range of options should be available for people according to their preferences and needs [21].

### New approaches to enhance self-management

A recent systematic review of alternative models of care found multifactorial individualized telehealth and community- or home-based CR were effective alternatives as they have produced similar reductions in CVD risk factors compared with hospital-based programs [22]. This echoes findings from other reviews of home-based CR [23] and telehealth [24]. There is a paucity of research describing the effectiveness of alternative models of CR in rural, remote, and culturally diverse populations. However, evidence suggests that hospital-based strategies may not be able to deliver effective CR to these populations. Local healthcare systems may need to integrate alternative models of CR, such as brief interventions tailored to individual’s risk factor profiles, as well as community- or home-based programs, to ensure that choices are available that best fit patient’s needs, risk factor profile, and preferences.

### The potential of mobile phone delivered self-management

While telehealth [24] and Internet-based programs have been shown to be effective, they are limited due to predominant reliance on desktop and landline communication, whereas technology is now more mobile. Mobile phones are the most common device for communication worldwide—including developing countries—and are used to deliver behavioral-change programs and improve disease self-management [25]. Mobile phones have potential to influence behavior at a population level because the technology is widely available globally, inexpensive, and allows instant delivery of information [26].

Text-messaging—or short message service (SMS)—is the most widely used mobile phone intervention. Two systematic reviews [27, 28] support the effectiveness of SMS interventions across a range of risk behaviors (e.g., smoking) and chronic conditions (e.g., diabetes and asthma). A 24-week text-messaging intervention in the HEART trial increased walking and leisure time exercise in people who were post ACS (*n* = 171), but did not increase maximal exercise capacity [29, 30]. The TEXT ME trial—which is the largest trial in people with CVD (*n* = 710) [31]—reported statistically significant positive effects on low-density lipoprotein (LDL) cholesterol, and sizeable effects on secondary outcomes such as blood pressure and physical activity. This intervention involved delivery of regular semi-personalized text messages providing advice, motivation, and information that aimed to improve diet, increase physical activity, and encourage smoking cessation. While this study represents a good initial evaluation of a text messaging intervention for enhancing CHD outcomes, future work is still required. First, it was conducted in a single center in Australia with many participants excluded due to language barriers or not owning a mobile phone, which limits its generalizability. Second, the intervention was evaluated as a stand-alone strategy, thus it was unclear whether the intervention was more or less beneficial to those in traditional programs. Third, implementation of the intervention in a real-world setting was not assessed in the study.

We previously conducted the Text4Heart randomized controlled pilot trial (*n* = 123) [32, 33], of a 6-month theory-based program of daily text messages and a supporting website in addition to usual CR services. Using a composite measure of lifestyle change (exercise, diet, smoking, alcohol), we observed a significant treatment effect on adherence to lifestyle behaviors at 3 months (adjusted OR = 2.55, 95% CI 1.12 to 5.84; *p* = .03), but not at 6 months (adjusted OR = 1.93, 95% CI 0.83 to 4.53; *p* = .13). At 6 months, the intervention group had greater self-reported medication adherence (mean difference in scores: 0.58, 95% CI 0.19 to 0.97; *p* = .004), with 51% reporting high adherence compared to 32% in the control condition. Text4Heart was also associated with lowered LDL cholesterol at 6 months compared with control (mean difference: − 0.25, 95% CI -0.49 to 0.003; *p* = .05). Participants reported high fidelity to the text-message component of the intervention, with 85% of intervention participants reading all their messages. Text4Heart was well-received with 84% of participants reporting that the program helped them recover, and 90% of participants would have recommended it to others who had a cardiac event [33]. A definitive trial of Text4Heart to determine its effectiveness using an objective measure is now required to determine the sustained effect of the intervention for augmenting existing cardiovascular services.

### Aims

To determine the effectiveness and cost-effectiveness of the Text4Heart II self-management program—in addition to usual care—to improve adherence to medication and lifestyle change in people with an ACS. A second aim is to use the RE-AIM framework [34] to inform the potential implementation of the Text4Heart II program to augment existing CR services with two district health boards in Auckland, New Zealand.

### Hypotheses

1. Text4Heart II will improve self-management of ACS, as seen by increased adherence to medication and lifestyle behaviors at 6 and 12 months compared to standard CR care alone,
2. Text4Heart II will be cost-effective, and
3. Text4Heart II will be imminently scalable for roll out within the existing healthcare system in New Zealand (district health boards)

## Methods

**Fig. 1:**
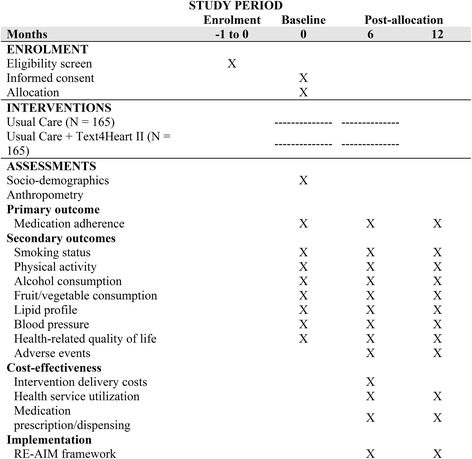
Schedule of enrollment, interventions, and assessments (Standard Protocol Items: Recommendations for Interventional Trials (SPIRIT) Figure)

The Text4Heart II trial is a two-arm, parallel, superiority RCT conducted in two large metropolitan hospitals in Auckland, New Zealand. The study protocol is in accord with the Standard Protocol Items: Recommendations for Interventional Trials (SPIRIT) 2013 Statement [35], and was prospectively registered in the Australian New Zealand Clinical Trials Registry on 1 April 2016, (ACTRN12616000422426). The intervention is described according to the Consolidated Standards of Reporting Trials (CONSORT)-eHealth Checklist [36]. The trial schedule is presented in Fig. 1, and the SPIRIT Checklist is reported in Additional file 1.

### Study population and recruitment

Adults who are clinically stable, able to read English, and provide informed consent are invited to participate into the study either while inpatients, or shortly after discharge following ACS or post percutaneous coronary intervention. Exclusion criteria include untreated ventricular tachycardia, severe heart failure, life-threatening co-existing disease with life expectancy below 1 year, and significant exercise limitations other than CVD. Potential participants will be given information sheets by a researcher and informed consent will be obtained either in writing, or verbally if the participant is already discharged.

All patients admitted with ACS or who undergo angiography are registered in the All New Zealand Acute Coronary Syndrome Quality Improvement register (ANZACS-QI) [37]. ANZACS-QI collects detailed information on risk factors, diagnosis, investigations, as well as management and complications during admission, and is embedded in > 90% of hospitals in New Zealand. Data are able to be linked to laboratory results and dispensing from chemists. Anonymized linkage to patients’ unique National Health Index number allows data to be obtained on mortality and rehospitalization for subsequent analysis [38]. This study design, utilizing the strengths of the clinical registry combined with the specific clinical trial protocol, provides a unique opportunity to collect study data at low cost and with no additional participant burden.

### Sample size

A total of 330 participants (165 per group) will provide 80% power at the 5% level of significance (two-sided) to detect an absolute difference of 15% between the two groups, in the proportions of participants adherent to medication 6 months after randomization (assuming a control rate of 30%). This is a conservative control rate and is based on our self-reported Text4Heart pilot data, and New Zealand research that found only 60% of patients had a Medication Dispensing Ratio (SDR) > 0.8 for statins only [39]. This value is likely to be lower when all classes of medication (statins, antihypertensive, and antiplatelet therapies) are considered. If the control rate was indeed 60% then a total of 304 participants would be required to detect an absolute difference of 15%; thus, we would be adequately powered with our proposed sample size.

### Randomization, allocation concealment, and blinding

Upon completion of baseline assessment a researcher will randomly allocate eligible participants at a 1:1 ratio to the intervention or control arm, using blocked (variable block sizes, 2 or 4) stratified (hospital site) randomization. The allocation sequence will be computer generated by an independent statistician not involved with trial conduct, and concealed by a centralized computer system that will reveal treatment allocation only after submission of baseline data. Study investigators (but not participants) are blinded to intervention allocation throughout the trial. The primary outcome, however, is derived from data linkage, which is blind to treatment allocation.

### Intervention and control

All participants will receive usual care, which includes CR support. In addition, those allocated to the Text4Heart II intervention arm will receive a personalized, automated program of CR delivered via text message over 24 weeks. The overall goals of the intervention are to encourage and promote adherence to medication, healthy diet, stress management, regular exercise, reduced alcohol consumption, and smoking cessation (if applicable). Participants will be able choose additional focussed intervention modules at baseline that address risk factors that they identify as most relevant to them, such as physical activity, heart healthy diet, stress management, and smoking cessation.

Participants receive one message per day for the first 12 weeks, a technical support phone call at 12 weeks, and five messages per week for the remaining 12 weeks. Messages will be personalized (including participants’ names) and sent at times to suit participants. The intervention is predominantly unidirectional but participants will be able to reply to text messages and a researcher will answer within 24 h. All participants will be offered brief training at enrollment on how to read, delete, and save text messages. Messages are categorized into four groups (see below). Non-smokers receive one to two general heart health messages, one to two physical activity messages, and one to two dietary messages per week. Smokers receive one general heart health message, one to two physical activity messages, one to two dietary messages, and one to two smoking-cessation support messages per week.

At registration, intervention group participants will select their preferred receipt times for educational (early morning, late morning, early afternoon, late afternoon, or evening) and medication reminder messages to ensure that the timing is appropriate for their needs. Details of the text-message content are provided below.

### Intervention content

#### General heart health and medication adherence

General health information messages that include facts about risk factors and medication will be provided. Messages will include information and strategies to help participants adhere to their prescribed medication regimen—including information on the value of taking medication to reduce recurrent events and hospitalization, reminders to have a regular check-up with their physician, enhancing self-management, and addressing illness perceptions and medications beliefs. In addition, advice will be given about contacting their physician if unwell, practical tips on how to improve lifestyle though habit formation and environmental prompts, and how to enlist support from others.

#### Physical activity

Participants will receive messages derived from the successful HEART trial [29, 30], that address the importance of being physically active. Message content will include suggested activities, key strategies to enhance uptake and maintenance of physical activity (e.g., goal setting, self-monitoring), and a generic exercise prescription that suggests the type, frequency, duration, and intensity of exercise.

#### Dietary behavior

Participants will be supported to reduce dietary saturated fat and salt intake, and to manage their weight. All content was developed, pre-tested and successfully piloted prior to the Text4Heart II trial [40]. Participants will receive text messages promoting healthy eating strategies, advice on choosing healthy food, and food preparation. The messages focus on supporting behavior-change strategies.

#### Smoking cessation

Participants who smoke tobacco will receive components of the successful STOMP text-messaging smoking-cessation intervention [41–43]. They will be sent regular messages providing smoking-cessation advice and support (e.g., symptoms to expect on quitting, tips to avoid weight gain and to cope with craving, advice on avoiding smoking triggers).

All messages are grounded in established psychological (Common Sense Model) [44] and behavior-change (Social Cognitive) theory [45] and will focus on modifying perceptions of the symptoms, timeline, causes, consequences, understanding of, personal control over, and ability of treatment to prevent, CVD [46], as well as altering the key mediators of behavior change including self-efficacy, social support, and motivation.

### Outcomes

All outcomes are assessed at 6 and 12 months post randomization. The primary outcome is the proportion of participants adhering to medication at 6 months. Medication adherence is defined as SDRs of 80% for statins, antihypertensive, and antiplatelet therapy classes of medication (calculated separately)—consistent with guideline-recommended therapy [47]—where SDR is calculated as the number of days that the supply is obtained divided by the number of days in the observation period. Participants’ community pharmacy dispensing records will be linked using their unique National Health Index number via the National Pharmaceuticals Collection database. This approach has been used successfully in New Zealand to assess statin use [39]. To adjust for days not covered due to death or days spent in hospital these periods are subtracted from the 6-month coverage time. The number of days supplied will be estimated from strength per unit and daily dose variables summed from pharmacy claims during the observation period. To account for any previous supply of the medication (before discharge), medication claims in the 3 months prior to admission are collected. The SDR for each class of medication (statins, antihypertensive, and antiplatelet therapies) will be recorded as secondary outcomes.

### Secondary outcomes

In a similar manner to the primary outcome, the proportion of participants adhering to medication will be assessed at 12 months. Self-reported outcomes will be measured by a trained research assistant during a telephone call at 6 and 12 months. Self-reported medication adherence will be assessed using the Morisky 8-item Medication Adherence Scale [48]. As per the Text4Heart pilot trial [32, 33], adherence to recommended lifestyle behaviors will be measured using a composite health behavior score adapted from the EPIC-Norfolk Prospective Population Study [49]. The following measures will be used to determine participants’ health behavior scores:

1. Smoking status will be measured using three items from a validated smoking history questionnaire [50] including whether participants have ever smoked, have had a puff of tobacco in the last week and when they quit smoking (if appropriate)
2. Physical activity level will be assessed using the Godin Leisure Time Physical Activity Questionnaire (GLTPAQ) [51]. This simple, three-item questionnaire has well-established reliability and validity and has been used in patients undergoing CR
3. Alcohol consumption will be measured using the Alcohol Use Disorders Identification Test alcohol consumption questions (AUDIT-C) [52]—a screening tool designed to assess units of alcohol consumed per week, and identify people who are hazardous drinkers. Index cards referencing standard drink sizes will be used to reduce comprehension errors
4. Fruit and vegetable intake are assessed by two New Zealand-specific questions used in the 2006/2007 New Zealand Health Survey (*n* = 12,488, including adults with CHD) [53]

Participants receive a score on a 4-point scale for each of the four key risk factors, with 1 point each assigned for being a current non-smoker, meeting physical activity guidelines to achieve some health benefits (defined as ≥ 14 units on the GLTPAQ), consuming ≤ 14 standard units of alcohol per week, and consuming at least five servings of fruit and vegetables per typical day.

Using participants’ encrypted National Health Index identification numbers we will be able to obtain clinical data (lipid profiles and blood pressure) from the ANZACS-QI database to maximize collection of these outcomes. Data describing hospital events, clinical information, as well as medication prescribing and dispensing will also be captured. For the purpose of this trial we will access data on lipid profile (total/LDL/HDL cholesterol) and blood pressure, from admission and routine follow-ups. Using ANZACS-QI we will also be able to access information on readmissions and related hospital events.

Intervention delivery costs—including text-message service and per message costs as well as health service staff time for recruitment and program facilitation—will be collected. Any changes in health service utilization observed between intervention and control groups will lead to an estimation of the costs of those changes with the assistance of District Health Board funding information analysts.

### Adverse events

All participants will continue with their usual care for ACS. No individual clinical advice is given through the pre-programmed text messages. We will be evaluating hospitalizations or health service utilization as part of the outcomes specified above.

### Statistical analysis

Trial data collected from all eligible participants will be linked with the national ANZACS-QI database using participants’ encrypted National Health Index number for the purpose of analysis. Treatment evaluations will be performed on the principle of intention-to-treat (ITT). Missing data on the primary outcome will be considered as non-adherence in the ITT approach. Sensitivity analyses will be conducted to test the robustness of main findings using different assumptions on the missing data if the proportion of missing exceeds 10%. The proportion of participants adhering to medication at 6 months, with or without intervention, will first be summarized as frequency and percentage. Logistic regression will be conducted to evaluate the main treatment effect (OR and 95% CI), adjusting for pre-defined baseline prognostic factors. For all secondary outcomes collected at 6 and 12 months post randomization, generalized linear regression models will be used to test the effect of intervention between two groups, using a link function appropriate to the distribution of outcomes. More specifically, an identity link will be used for continuous outcomes under normal distribution and a logit link for binary outcomes under binomial distribution. Regression models will adjust for baseline outcome value (where collected) and stratification factor. Model-adjusted estimate (mean difference for continuous outcomes and OR for binary outcomes) will be reported at each scheduled visit, with 95% CI and associated *p* value. Missing data will not be imputed on secondary outcomes without adjustment for multiple testing. Statistical analysis will be performed using SAS version 9.4 (SAS Institute Inc., Cary, NC, USA). All statistical tests will be two-sided at the 5% significance level.

#### Cost-effectiveness analysis

This analysis will adopt a health system perspective. We will use the EQ-5D—a generic and validated measure of quality of life for which reliable New Zealand population preference values are available—to obtain a single preference index for calculation of Quality-adjusted life-years (QALYs) to assess cost per QALY, for comparison with other programs. The incremental cost of making one extra participant adherent to CR using the intervention compared to usual care will be calculated. Ninety-five percent confidence intervals for incremental cost-effectiveness ratios, 95% confidence ellipses on the incremental cost-effectiveness plane, and cost-effectiveness acceptability curves will be calculated to compare the intervention with usual care. Markov modeling will combine these data with other information from a systematic review of cost-effectiveness studies of CR to identify the long-term cost-effectiveness of the intervention.

### Data management

Data will be entered into an electronic collection system provided by Enigma Solutions which will be linked to the ANZACS-QI register. Range checks will be implemented and 10% of data will be checked for consistency against source data.

### Evaluating implementation

We propose using the RE-AIM model proposed by Glasgow and colleagues [34, 54] to determine uptake and sustainability of the intervention. The RE-AIM framework—which emphasizes collecting information about the Reach, Effectiveness, Adoption, Implementation, and Maintenance of an intervention—is an evaluative framework for guiding the evaluation and reporting of health intervention effectiveness [55]. Further, RE-AIM provides a framework for determining which programs work under real-world conditions and which programs should be sustained. The RE-AIM framework is an ideal tool to use as the basis for planning and evaluating the success of mobile phone self-management interventions [56, 57]. It also aligns with systems-based approaches and allows for assessment of vertical (e.g., adoption decisions within a given organization) and horizontal (e.g., adoption across different sectors) components [58]. The RE-AIM framework includes both individual- and setting/staff-level variables. Two dimensions operate at the individual level (reach and effectiveness).

A mixed-methods approach [59] will be used to assess the key components of RE-AIM. Qualitative and quantitative data will be combined to thoroughly understand the extent to which the self-management intervention could be successfully implemented within the New Zealand health context [60]. To achieve this, data will be collected on each RE-AIM dimension as proposed by Kessler et al. [61] and Glasgow [60]. Some of these data will be collected as part of the trial (e.g., data to inform reach, effectiveness and costs/resources). Additional data will be collected specifically for the RE-AIM analysis (detailed below).

To determine intervention reach, we will assess the number of people who participate as a proportion of those who are eligible, compare the characteristics of those who do and do not participate, and provide detailed information of reach and recruitment issues [62, 63]. To achieve this, all potential participants approached about the study will be screened and basic information collected. Those who decline participation will have their information permanently de-identified. Screening information will be used to determine the representativeness of those who agree to participate compared to those who decline. Qualitative data will be gathered from those who decline about their reasons for choosing to not participate. For those who agree, a brief semi-structured interview will be conducted to determine their perspectives of the proposed intervention, their current self-management behaviors, and any concerns they might have that would have impacted on their involvement or adoption.

In addition to the primary outcome, biological-, psychosocial-, demographic-, and program-specific parameters will be assessed as potential moderators of intervention effectiveness.

RE-AIM dimensions Adoption, Implementation, and Maintenance will be assessed from both provider and organizational perspectives [54]. Key informant interviews will be conducted with stakeholders (medical practitioners and allied health staff) to obtain qualitative data from providers across these three RE-AIM dimensions, to determine factors that may enhance organizational adoption and maintenance, and potential adaptations to heighten the likelihood of consistent delivery across providers and locations. Quantitative assessment of implementation will be determined by measuring text-message responses where appropriate. Maintenance at the organizational level will be determined by conducting key informant interviews with stakeholders (e.g., chief executive officers) as well as government and non-government agencies (Ministry of Health, Heart Foundation) examining potential sustainability options for each organization [64]. To evaluate Maintenance at the patient level, patient outcomes will be measured at 12 months via ANZACS-QI. Long-term attrition (%) and differential rates by patient characteristics or treatment condition will be examined. At the organizational level, we will collect data on whether the interventions were sustained at more than 6 months post study funding, or which elements were retained after the program was funded.

Qualitative data collection and analysis will be conducted by a trained and experienced researcher. Interviews will be digitally recorded and transcribed. Data will be entered using NVivo software to enable qualitative analysis. An inductive analysis approach will be used to identify the key themes to emerge from the data. These data will be collated according to each domain of the RE-AIM framework. These data sources will be combined and—together with the advisory group—investigators will make recommendations to determine the extent to which the self-management intervention achieved the desired RE-AIM outcomes. This approach has been successfully used with other behavior-change interventions and will also be valuable for informing optimal scenarios for funding and implementing this self-management program [54, 65, 66].

## Discussion

The Text4Heart II trial will determine the effectiveness of a text-message-based intervention in addition to usual care for improving adherence to medication and lifestyle behaviors at both 6 and 12 months. Using the RE-AIM framework this trial will provide much needed data and insight into the potential implementation of Text4Heart II to augment existing cardiac services within two major metropolitan hospitals. The protocol, in accordance with the SPIRIT Statement, includes outcomes from recent systematic reviews of mobile health with the aim of adding quality evidence to the body of academic literature.

This trial addresses many limitations/criticisms of previous mHealth trials [43, 67]; it builds on the Text4Heart pilot trial, is adequately powered, has sufficient duration to elicit behavior change, and the follow-up assessments (6 and 12 months) are long enough to determine the sustained effect of the intervention. We also outline the behavior-change theory used, and intervention content, which will enhance its replicability. Using data linkage (National Pharmaceuticals Collection and ANZACS-QI registries) our Text4Heart II trial will not only provide an objective measurement of medication adherence, but will be one of the first of its kind to provide much needed data on sustained effects on clinical outcomes including hospitalization and mortality. While mHealth is often touted as a low-cost intervention that can be delivered at scale, few studies provide evidence of this. The Text4Heart II trial will also provide much needed data on the cost-effectiveness of this approach, and its potential implementation and scalability as a national service.

In summary, Text4Heart II should produce new knowledge on the effectiveness and cost-effectiveness of an innovative and promising mHealth program to improve self-management of heart disease. It extends previous research by investigating the sustained effects of a text-message intervention, and will offer unique insights into clinical effects. If effective, this approach could substantially reduce deaths and hospital admissions in a group of patients who account for up to one third of all hospital admissions. We will provide much needed insight into the potential of implementing this program at a national level, thereby augmenting existing CVD service delivery.

### Trial status

Recruitment for the Text4Heart II trial opened in July 2016 at Auckland City and North Shore Hospitals (Auckland, New Zealand). Recruitment is currently open, and expected to be completed in October 2017. The original study protocol was finalized on 18 March 2016; this manuscript reports version 5, amended on 13 December 2016. The primary amendment extended follow-up to 12 months, facilitated by an award from National Heart Foundation of New Zealand. Version control has been implemented to document all amendments to the study protocol, and these will be communicated to the Ethics Committees and trial investigators as required.

## Additional file

Additional file 1: SPIRIT Checklist. (DOC 120 kb)

## Abbreviations

ACS: Acute coronary syndrome
ANZACS-QI: All New Zealand Acute Coronary Syndrome Quality Improvement register
AUDIT-C: Alcohol Use Disorders Identification Test
CHD: Coronary heart disease
CR: Cardiac rehabilitation
CVD: Cardiovascular disease
GLTPAQ: Godin Leisure Time Physical Activity Questionnaire
ITT: Intention-to-treat
LDL: Low-density lipoprotein
MI: Myocardial infarction
OR: Odds ratio
QALY: Quality-adjusted life-year
SDR: Medication Dispensing Ratio
SMS: Short message service
